# Brave new world: expanding home care in stem cell transplantation and advanced therapies with new technologies

**DOI:** 10.3389/fimmu.2024.1366962

**Published:** 2024-04-26

**Authors:** Iñigo Romon, Soledad Gonzalez-Barrera, Carmen Coello de Portugal, Enrique Ocio, Isabel Sampedro

**Affiliations:** ^1^ Transfusion Section, Hematology Department, University Hospital “Marques de Valdecilla”, Santander, Spain; ^2^ Home Hospitalization Department, University Hospital “Marques de Valdecilla” - Instituto de Investigación Valdecilla (IDIVAL), Santander, Spain; ^3^ Transfusion Service, Toledo University Hospital, Toledo, Spain; ^4^ Hematology Department, University Hospital “Marques de Valdecilla” - IDIVAL, Santander, Spain

**Keywords:** stem cell transplantation, hospital at-home, outpatient, safety, training, drones, wearables, artificial intelligence

## Abstract

Hematopoietic stem cell transplantation and cell therapies like CAR-T are costly, complex therapeutic procedures. Outpatient models, including at-home transplantation, have been developed, resulting in similar survival results, reduced costs, and increased patient satisfaction. The complexity and safety of the process can be addressed with various emerging technologies (artificial intelligence, wearable sensors, point-of-care analytical devices, drones, virtual assistants) that allow continuous patient monitoring and improved decision-making processes. Patients, caregivers, and staff can also benefit from improved training with simulation or virtual reality. However, many technical, operational, and above all, ethical concerns need to be addressed. Finally, outpatient or at-home hematopoietic transplantation or CAR-T therapy creates a different, integrated operative system that must be planned, designed, and carefully adapted to the patient’s characteristics and distance from the hospital. Patients, clinicians, and their clinical environments can benefit from technically improved at-home transplantation.

## 1 Introduction

Medicine strives to find the best place of care for patients, which can be defined as one that achieves the best clinical outcomes with minimal disruption for the patient, at a manageable cost (1, 2).

With the number of hematopoietic stem cell transplantations (HSCTs) increasing, due to the number of conditions treated and the incorporation of low and middle-income countries into the HSCT field, rising costs, staffing difficulties and hospital capacity shortages must be addressed. The Worldwide Network for Blood and Marrow Transplantation identified the major cost drivers as patient characteristics, laboratory and radiology, drugs, supportive care, quality management and staff training, and graft procurement (3).

Outpatient (OP) and at-home management, were adopted early during the inception of hematological therapies, including HSCT (4).

The advent of CAR-T platforms with an increased range of indications and more limited toxicity, together with the physician’s experience and confidence, has increased interest in early patient discharge while maintaining close patient monitoring (5).

Social trends, cost constraints, and the impact of COVID-19 (6) support the shift from inpatient (IP) care to more acceptable modalities, like home care for HSCT or CAR-T cell therapies (2).

However, safety concerns for outpatient modalities will always be extremely important until HSCT and cell therapy procedures cause less aggressive effects on the patient’s health.

Fortunately, emerging technological developments (i.e., wearable devices, artificial intelligence (AI), facial recognition) provide important opportunities to increase safety while reducing costs. This mini-review discusses HSCT and CAR-T as models for clinical out-of-hospital management and takes a prospective view of technologies that can help maximize clinical efficacy, contain costs, and enhance patients´ well-being.

## 2 Outpatient and at-home HSCT

In 1997, Jagannath reported 91 multiple myeloma autologous HSCT (auto-HSCTs) followed on an OP basis (7).

In the OP setting, patients receive the conditioning regimen and HSCT infusion at an outpatient clinic or the hospital, and are then discharged and followed up in an ambulatory day clinic.

Nowadays, several reports on the management of HSCTs on an OP basis have been published, but systematization and research are still lacking (8). Even the definition of OP setting can be controversial since several modalities exist, from OP to full at-home management (9).

Holbro (10) reported 91 auto-HSCTs in myeloma patients managed entirely at an OP clinic where 84% had to be readmitted during the first 100 days, mostly for febrile neutropenia. No patient died or required ICU care. Savings of nearly 20,000 Canadian dollars per patient were calculated compared with IP HSCT.

McDiarmid (11) retrospectively analyzed infections in 178 allogeneic (allo-HSCT) and 508 autologous OP HSCTs from 1,045 consecutive allo- and auto-HSCTs. The infection incidence was significantly lower in both OP auto- and allo- cohorts compared to IP setting. The 100-day non-relapse mortality (NRM) was significantly lower for OP allo-HSCT than for IP allo-HSCT. They concluded that OP allo- and auto-HSCT had comparable short-term outcomes and complications to IP HSCT.

Granot (12) retrospectively reported 1,037 OP allo-HSCT, and noted that 47% of OP patients were never hospitalized as well as having a lower 5-year risk of NRM than IP HSCT (13% OP vs. 23% IP, p<0.001). OPs evaluated the experience favorably. Shingal (13) reported 856 OP allo-HSCT (a third of whom were never hospitalized) with a median decreased stay of 6 days but no report on long-term results was made.

A Mexican group found that performing OP allo- and auto- HSCTs, with simplified stem cell conservation or conditioning regimens, amongst other measures, could be a solution for countries needing less resource-intense modalities (14, 15).

In the at-home model, patients equally receive the conditioning regimen and stem cell infusion at the hospital or outpatient clinic, spending the aplastic phase at home, where they are cared for by a healthcare team and only returned to hospital if severe complications appear.

A group in Barcelona (16, 17) performed 50 auto-HSCT at home, suggesting nearly 50% cost reduction with great patient satisfaction. Gonzalez-Barrera (18) reported 84 at-home auto- HSCTs with a median at-home stay of 17 days, 86% experienced neutropenic fever, 44% presented grade 3-4 mucositis, and 26% received parenteral nutrition. Hospital readmission occurred in 12% of patients, usually for sepsis, and lasted a median of 9 days, with no transplant-related mortality (TRM). The Durham group analyzed both auto- and allo-HSCTs, finding similar results and good preservation of quality of life for at-home HSCTs (19) compared to IP controls.

Svahn (20) analyzed at-home allo-HSCTs finding significantly reduced TRM (13% at-home vs. 44% IP controls), with improved four-year survival (63% at-home vs. 44% IP controls). They suggested that this advantage could be due to better oral nutrition, resulting in the reduced probability of acute graft-versus-host disease (GvHD) and bacteriemia (21). Pediatric at-home allo-HSCTs has also been performed with favorable results (22, 23). Jenkelowitz reported great caregiver satisfaction for at-home HSCT (24)

Gonzalez (8) summarized 29 auto- and allo-HSCT studies, finding evidence of improved health outcomes, quality of life, and at-home/OP models vs IP effectiveness. Overall survival rates comparing both models were similar for both autologous and allogeneic patients. They also found that OP and at-home HSCT were safe, perhaps more than conventional modalities. They estimated a shorter average stay with the OP/at-home models, being 55% and 19% shorter than the IP model for auto- and allo-HSCTs, respectively, which could ease hospital bed shortages and waiting times. The average cost reduction over the IP-HSCT model was 33.42% and 19.27% for auto- and allo-HSCTs, respectively (Appendix 1 and 2 update a published summary of the studies on allo -HSCT and their results).

As Gonzalez has indicated (8), the high-quality research in the field that is needed for evaluation and benchmarking is lacking. Research should include clinical trials with uniform defining criteria for the different HSCT care models, uniform cost analyses, and assessment of personal experiences and quality of life of staff and patients involved. Finally, the different HSCT modalities should be registered in the different HSCT databases.

## 3 Outpatient CAR-T therapies

In the OP CAR-T model, lymphodepletion, and CAR-T infusion are administered at an OP clinic, with patients staying at home and followed up at fixed intervals at the OP clinic, and admitted to hospital preemptively, or to monitor or treat adverse events (25, 26).

Several authors have reviewed the challenges and requirements for OP CAR-T (5, 27–29).

Borogovac (26) reported 21 patients treated at a CAR-T unit designed for the OP setting. Seventy-one percent were admitted after discharge to treat adverse effects and stayed a median of 8 days, but none needed ER or ICU care. None died. Similar experiences were reported by McGann (25) with 40 patients. Cytokine release syndrome (CRS) and immune effector cell-associated neurotoxicity syndrome (ICANS) had a similar incidence as with IPs (25, 26). **Table 1** summarizes these findings.

**Table 1:** Reported experiences of CAR-T outpatient management in daily practice.

Author (reference)	Borogovac (26)	Mc Gann (25)
Year	2022	2022
Study duration	2 years	3 years
Visit schedule after CAR-T infusion	Daily, days 1 – 14 Three times/week, days 15 – 28	Daily
Inpatient management, n (‡)	2	8
Outpatient management, n	21	32 (*)
Type of CAR-T, n (%)	Axi 13 (62%) Tisa 6 (29%) Brex 1 (5%) Liso 1 (5%)	4-1BB construct 25 (78%) CD28 construct 7 (22%)
Patients readmitted after CAR-T infusion, n (%) At 72 hours At 30 days	5 (24%) 15 (71%)	27 (84.5%) 28 (87.5%)
Days to readmission after CAR-T infusion, median (range)	4 (1-21)	Not stated
Days of stay after readmission, median (range)	8 (1-30)	14 (9-52)
Cause for readmission, n (%) Fever Neurological symptoms CRS	13 (87%) 2 (13%) 12 (57%)	12 (38%) 12 (38%) 25 (78%)
Adverse events grade > 3 CRS, n (%) Neurotoxicity, n (%)	1 of 12 (8%) 1 of 6 (17%)	1 of 25 (3%) 5 of 12 (45%)
Treatment of adverse events Tocilizumab, median number of doses (range) Dexametasone, days (range)	1 (1- 5) 2 (1 – 21)	Not stated Not stated
ICU or emergency use, n (%)	None	3 (9.5%)
Deaths	None	3 (9.5%) at 90 days
ORR % (type of response)	Axi 76% (9CR, 1PR) Tisa 100% (5 CR, 1 PR) Brex 100% (1CR) Liso 0 (progression)	81% at 30 days (20 CR) 72% at 90 days (20 CR) (**)

(‡) Those patients received their CAR-T as inpatients and remained hospitalized during all their clinical course, as they were not considered suited for outpatient management.

In the outpatient management (OP), patients receive lymphodepleting drugs and CAR-T infusion in the ambulatory day clinic; some are directly readmitted, while the rest are followed up at an ambulatory day clinic unless readmission is deemed necessary.

(*) A group of patients in this study were initially managed as OP, and were admitted preemptively to monitor or treat adverse effects, others were admitted only if clinically indicated.

CAR-T, chimeric antigen receptor – T; Axi, axicabtagene ciloleucel; Brex, brexucabtagene autoleucel; Tisa, tisagenlecleucel; Liso, lisocabtagene maraleucel; CRS, cytokine release syndrome; ICANS, immune effector cell-associated neurotoxicity syndrome;ICU, intensive care unit; ORR, overall response rate; CR, complete remission; PR, partial remission.

(**): global data, not segregated by the type of CAR-T infused.

Paludo (30) reported 123 OP CAR-T patients managed with a remote patient monitoring program (RPM), to monitor vital data and neurological symptoms (31). Sixteen percent could be completely managed in the OP setting, with a median stay of 8 days for those who were hospitalized to treat post CAR-T adverse effects, with 6% needing ICU. Most patients (83%) were satisfied with their RPM. Dwivedy (32)Shao (33) and Bachier (34) reported similar experiences. Appendix 3 summarizes these reports.

## 4 At-home and outpatient procedures: practical considerations

At-home/OP HSCT is a well-recognized management platform (9). Borogobac (26) defined the key components of an OP CAR-T model: create a multidisciplinary team, train nursing staff, educate community providers, increase patient knowledge and support, acquire physical space for CAR-T operations, adhere to standardized procedures and processes, review procedures, outcomes and finances. Similar considerations were made by an expert panel of the American Society of Transplantation and Cellular Therapy (28)

At-home/OP programs should define patient eligibility criteria, patient and caregiver information, and training. Appendix 4 summarizes and updates prior published provisional patient acceptance criteria for at-home/OP HSCT and CAR-T therapy programs.

A well-trained staff with on-call nurses and physicians to attend routine or emergency calls as well as an antibiotic policy adapted to the at-home/OP context and efficient transport for diagnostic samples are needed. Both routine and emergency operational scenarios must be assessed (8, 9).

Allo-HSCT presents a higher incidence of infections, including aspergillosis, and adverse events like GvHD or acute renal failure (20, 35), which should be detected and managed during the early stages. Common complications of HSCT [fever, mucositis (36, 37), GvHD, capillary leak syndrome, bleeding, diarrhea, nutrition (21)], or CAR-T (CRS, ICANS) should have defined prevention and treatment strategies, which must be planned and trained for. Decreased physical performance should be addressed with a specialized exercise program (38, 39).

Interventions (i.e., fluid therapy, transfusions, antibiotics, catheter care) should be designed to minimize clinical team visits, considering the distance of patients’ homes from the hospital. For example, the use of continuous infusion pumps (40) or self-administration could be contemplated (41). For OP CAR-T, special protocols for the early use (even while at home) of tocilizumab or dexamethasone in the treatment of complications should be implemented.

**Table 2** summarizes critical issues when implementing an at-home HSCT/OP CAR-T program.

**Table 2:** Summary of critical checkpoints for at-home/OP HSCT or CAR-T therapies (8, 18, 26, 28).

Quality and regulation	Team	Patient & home	Caregiver	Pre-procedure	Procedure	Post-procedure	Records & data
• Are there national / regional regulations applicable to at-home HSCT or cellular therapies? • How is the at-home / OP process designed? • What are the quality indicators? • How are incidents collected and analyzed? • Is there a quality auditing process in place? • How are changes validated?	• How is the at-home /OP team made up? Who is in charge? • Are staff resources sufficient? • If more than one hospital service is in charge of at-home / OP management, how do they interact? • How is the staff chosen and trained?	• How is the patient presented for at-home / OP treatment? • What are the inclusion / exclusion criteria? • How are selection criteria confirmed and checked? • Who informs the patient about at-home /OP procedure? • Who obtains the informed consent? How is it documented? • Who qualifies whether the patient´s home is adequate for HSCT / CAR-T? What are the criteria?	• Who qualifies the caregivers? • How are they trained? Who is in charge? • Who defines training?	• How are the conditioning / immunosuppressive /lymphodepletion regimes chosen? • How and where is conditioning / lymphodepletion administered? • Where are stem / CAR-T cells infused?	• How are clinical visits scheduled? • How are out-of-hours and emergencies attended? • How are materials delivered to the patients’ homes? • What is the infection prophylaxis policy? • How are clinical samples transported to the laboratory? • Can transfusions be given at the home? • How are common complications prevented and treated (infection, respiratory failure, diarrhea, nausea, vomiting, catheter malfunctioning, acute renal failure, CRS, ICANS)? • How are radiological tests performed? • Is there a specific antibiotic policy? • How is Graft vs Host Disease prevented, detected, evaluated, and treated?	• How are patients discharged from at-home / OP procedure?	• How are clinical data collected? • How are they transmitted to patients’ records? • How are data protected and stored?

HSCT, hematopoietic stem cell transplantation; OP, outpatient management, where patients receive lymphodepletion/conditioning regimen and infusion in the ambulatory day clinic or as inpatients; and are followed up at an ambulatory day clinic. CAR-T, chimeric antigen receptor T-cell; CRS, cytokine release syndrome; ICANS, immune effector cell-associated neurological syndrome.

## 5 Frontiers for at-home HSCT and CAR-T: improving patient safety and quality of care

Emerging technologies provide means for improved patient safety monitoring, which is of special concern for at-home/OP HSCT and CAR-T therapy programs. Remarkably, many can potentially feed data to patients’ records and form networks with AI, resulting in early warning systems and better patient records, creating a continuous care ecosystem.

a. Staff and caregiver training: at-home/OP HSCT and CAR-T programs require specific and timely training for both caregivers and health staff.

i. Clinical simulation (CS) is based on creating situations replicating common daily clinical problems with mannequins or actors which allows for ‘real life’ learning. Feedback and debriefing are provided by the supervisors (42). Simulation has been used successfully to train nurses and caregivers, resulting in improved learning performances, with training verification of the learning quality (43).
ii. Virtual reality (VR) is a real-world computer simulation that produces immersive images where the user communicates with the computer via an interface. VR, less expensive than simulation, has been used to improve caregiver and staff training in different contexts. This can result not only in improved skill uptake but also in stress reduction for caregivers (44, 45).

b. Patient electronics:

i. Facial recognition technologies (FRT): Cheng showed that oxygen saturation can be measured by remote photoplethysmography using a general-purpose camera (46). Due to its sensitivity, FRT can be more sensitive than clinician judgment, creating early warning systems and enabling improved clinical decision-making.

ii. Point of care tests (POC):

1. POC tests can reduce decision times and costs, waiving the time and complexity of sample or patient transport.
2. Glucose, coagulation, and urinary tests can be performed at home, often with one drop of blood or urine. Portable, miniaturized devices and nanotechnologies can perform leukocyte count or blood grouping (47–49).
3. Furthermore, implantable cytomimetic nanomaterials can detect bacteria in saliva, forewarning the introduction of a new, less invasive generation of clinical samples (50)

iii. Wearables and remote follow-up:

1. Patients can wear several sensor-carrying devices (hearing aids, smart clothes and watches, optical displays, subcutaneous sensors, electronic footwear, etc.) (51, 52). The sensors transmit data via different communication means (smartphones, Wi-Fi, routers, 5G networks…) to the healthcare team (51), giving more detailed patient supervision.
2. Those sensors can detect early deviations in patient health data (i.e., temperature, oxygen saturation, pH, lactate, respiratory rate, blood pressure, etc.), report accidents or guide behavioral therapy and rehabilitation, which is particularly useful for supervising physical activity during the HSCT process (53).
3. Medication delivery through wearable devices coupled with a skin sensor is commonplace for diabetes (54). The use of subcutaneous micro-needles could be particularly useful for drugs with a narrow therapeutic margin, like immunosuppressors (53).
4. In conclusion, wearables show great promise in providing a continuous flow of patient data and potentially delivering medication. Low-priced wearables could allow healthcare delivery, particularly to resource-strapped areas. However, many problems need to be resolved in technical (security, connectivity, precision, battery life, etc.), regulatory (privacy, clinical trials, data use), and clinical aspects (patient, caregiver, and staff training, data interpretation) before widespread use can be implemented. Recently, Hurtado (55) found that usability was considered acceptable and vital signs (heart rate, oxygen saturation, etc.) measured via a scanwatch had medium/high adherence, while temperature recorded via manual intervention had lower compliance. Recordings of quality-of-life assessment decreased during the study, and it was concluded that adding wearable devices to a telehealth clinical platform could potentially be synergistic for HSCT and CAR-T patient monitoring. Non-complete platform automation and the absence of a dedicated telemedicine team still represent major limitations, particularly for older patients and those with low digital education.

iv. Drones: unmanned vehicles (drones) are flying devices used for automated transport. Ground drones also exist.

1. Drones have been used to carry heavy weights, including blood for transfusion (56). They can fly at high speeds, sometimes faster than ambulances (57), being unaffected by bad traffic, and in this way reduce response times in routine and emergency settings.
2. Drones could deliver clinical materials (needles, syringes, tubing…), blood components, or drugs to patients’ homes or return samples to the hospital.
3. Many hurdles, like cost, user-friendly design, weight capacity or airspace control over cities, material traceability, accident liability, etc. remain.

v. Virtual assistants (VA) or AI virtual assistants are embodied or disembodied conversational agents that allow patients to ask questions, receive information, and report their clinical evolution to a technical system in a natural language.

1. With the rise of AI, VA are gaining ground as an aid in daily clinical information tasks. VA has been used to support home care for patients (58). VA has also been used with cancer patients (59).
2. Generally, patients and caregivers accept VA, which can both reassure them and increase medication compliance. Therefore, VA could be a valuable resource by supporting clinical teams with upfront patient evaluation and information during their daily work.
3. However, validation studies are still pending, and patient participation is critical when designing and implementing such services (59).

c. Artificial intelligence:

i. AI encompasses several technologies, from performing algorithms to neural networks that mimic the human brain and allow computer systems to emulate human intelligence and “learn” as they gather data (60), without the need for programming.
ii. AI can be used to support decision-making processes in HSCT, and refine predictive models of hematological diseases (61), potentially improving patient stratification. AI has been used in the HSCT context (62) to predict survival (63) or GvHD after HSCT (64). AI has the potential to help select the suitable drugs and doses (65) or support transfusion decisions (66, 67).
iii. During the HSCT/CAR-T process, AI can also be used in early warning systems, coupled with sensors or image devices. For example, AI coupled with FRT can detect patients’ vasovagal reactions (68).
iv. Many regulatory issues still need to be overcome, and a profound understanding of data analysis is required (60), with possible bias, ethical, and privacy issues to be solved before widespread uptake.

d. As technical advances improve communications and patient care, which in turn facilitate the implementation of at-home OP treatments; more diverse, patient- and process- centered organizational models will become necessary.

## 6 Conclusions: “brave new world”:

a. The feasibility of at-home HSCT is well established, and, in some instances, it could improve patient outcomes (infections, GvHD, survival), with reduced mortality (8, 16, 18, 20, 22). Outpatient management has been considered the future of CAR-T (5), with several dozens of patients already managed this way. However, those are usually single-center reports, so further research, including prospective comparative trials, is required to prove the advantages of at-home/OP management over IP treatment, in clinical and economic terms.

b. Safety and clinical efficacy are critical for at-home/OP HSCT and CAR-T operations. Clinicians must establish policies addressing the specific adverse events, adapted to the constraints of at-home/OP HSCT and CAR-T. An anticipatory approach must be taken, given the possible difficulties in managing patients outside the hospital setting. To this end, emerging technologies can create an improved ecosystem for HSCT/CAR-T.

i. Before HSCT/CAR-T:

1. AI applications can help physicians improve diagnostic accuracy, patient stratification, patient selection and the tailoring of conditioning regimens and prophylactic strategies.
2. Patients, caregivers, and clinical staff can be trained with VR or CS, with a more intense schedule, adapted to the requirements of at-home/OP HSCT and CAR-T programs.

ii. During HSCT/CAR-T:

1. VAs can collect information from patients and caregivers, supporting the work of clinical care teams.
2. Wearable sensors, coupled with AI platforms, enable constant patient supervision, resulting in early warning systems for adverse events (i.e., septic shock, hypoxemia).
3. Drones or POC devices allow simpler, faster care delivery.
4. Altogether, this allows for a more flexible working schedule for clinical care teams.

c. Technical support holds significant promise for at-home/OP HSCT and CAR-T programs. Gatwood proposed telemedicine and wearables as requirements for OP CAR-T management (5). However, even if high sensitivity and specificity levels are demonstrated, many issues linger: patient and caregiver acceptability, data safekeeping, training, regulation, etc.

i. Ethical concerns (i.e., privacy vs. continued surveillance) will have to be overcome via clinical trials.
ii. Clinical teams will have to validate the devices that provide the most significant information and learn the real value of the data received and adjust responses accordingly (e.g., physicians would have to learn to react to continuous oxygen saturation variations).

d. Staff education and training for routine and emergency responses must be an integral part of at-home/OP programs. Several barriers including psychological and personal factors, training opportunities or connectivity, as well as facilitators for the incorporation of digital technologies by healthcare staff should be addressed to implement real change (69).

e. Finally, at-home/OP HSCT and CAR-T therapies could result in improved clinical results and satisfaction rates for patients and staff over conventional hospitalization. Now is the time for further research. However, we should never forget that the ultimate objective of outpatient or at-home HCST and CAR-T programs is patient’s well-being (5), and not economics. The lessons learned in this demanding context could be expanded to the standard IP environment.

## Author contributions

IR: Conceptualization, Supervision, Writing – original draft, Writing – review & editing. SG: Investigation, Writing – review & editing. CC: Conceptualization, Investigation, Writing – review & editing. EO: Investigation, Writing – review & editing. IS: Conceptualization, Investigation, Writing – review & editing.
