# Supplementary material for: Brave new world: expanding home care in stem cell transplantation and advanced therapies with new technologies
Source: Front Immunol. 2024 Apr 26;15:1366962. doi: 10.3389/fimmu.2024.1366962 (PMC11082320; doi:10.3389/fimmu.2024.1366962)
Supplement: Supplementary file 1 [file Table_1.docx]

Appendix 1. Characteristics of studies focused on allogeneic transplants. This table **updates the table previously published by Gonzalez MJ (1)** The shaded row highlights an additional study by Singhal (2)

| **Author** | **Year, country** | **Study desing** | **Outpatient regime** | **Total patients (n)** | **Inpatient care model patients (n)** | **Outpatient care HaH (n)** |
| --- | --- | --- | --- | --- | --- | --- |
| Rizzo ** | 1999, USA | Nonrandomized prospective cohort study | Outpatient clinic | 132 | 115 | 17 |
| Svahn | 2000 ,Sweden | Single Center Prospective Case Control comparison | At home | 33 | 11 | 22 |
| Svahn | 2005, Sweden | Single Center Prospective Case Control comparison | At home | 90 | 54 | 36 |
| Nicolau | 2007, Brazil | Single Center Retrospective Case control comparison | Outpatient Clinic | 100 | 49 | 51 |
| Svahn | 2008, Sweden | Single Center Prospective Case Control | At home | 152 | 76 | 76 |
| McDiarmid * | 2010, Canada | Single Center Retrospective Case control comparison | Outpatient Clinic | 392 | 196 | 196 |
| Ringden | 2013, Sweden | Single Center Retrospective Case control comparison | At home | 292 | 146 | 146 |
| Granot | 2015, EUA | Single Center Prospective Case Control comparison | Outpatient Clinic | 1,037 | 548 | 489 |
| Cantu-Rodriguez * | 2016, Mexico | Single Center Retrospective Case control comparison | At home | 32 | 19 | 13 |
| Lisenko | 2017 , Germany | A retrospective single-centre analysis | Outpatient Clinic | 128 | 65 | 63 |
| Guru | 2019, USA | Single Center Retrospective Case control comparison | Outpatient Clinic | 151 | 116 | 35 |
| Gutierrez-Gracia | 2020, Spain | Single Center Retrospective Case control comparison | At home | 80 | 39 | 41 |
| Shingal | 2023, USA | Single Center Retrospective data analysis | Outpatient clinic | 853 |  | 853 |

*: This article analyses both allogeneic and autologous

**: Does not specify type of transplant.

1. González MJ, Urizar E, Urtaran-Laresgoiti M, Nuño-Solinís R, Lázaro-Pérez E, Vázquez L, Pascual-Cascón MJ, Solano C, Kwon M, Gallego C, et al. Hospital and outpatient models for Hematopoietic Stem Cell Transplantation: A systematic review of comparative studies for health outcomes, experience of care and costs. *PLoS One* (2021) 16:1–15. doi: 10.1371/journal.pone.0254135

2. Singhal S, Saadeh SS, Durani U, Kansagra A, Alkhateeb HB, Shah M V., Mangaonkar A, Kenderian S, Hashmi S, Patnaik M V., et al. Allogeneic Hematopoietic Stem Cell Transplantation in the Outpatient Setting: The Mayo Clinic Experience. *Transplant Cell Ther* (2023) 29:183.e1-183.e6. doi: 10.1016/J.JTCT.2022.12.016
