# Supplementary material for: Brave new world: expanding home care in stem cell transplantation and advanced therapies with new technologies
Source: Front Immunol. 2024 Apr 26;15:1366962. doi: 10.3389/fimmu.2024.1366962 (PMC11082320; doi:10.3389/fimmu.2024.1366962)
Supplement: Supplementary file 2 [file Table_2.docx]

| Appendix 2. Summary of results of health outcomes for allogeneic HSCT patients. This table **updates the table previously published by Gonzalez MJ (1)** The shaded row highlights an additional study by Singhal (2) | | | | | |
| --- | --- | --- | --- | --- | --- |
| **First Author** | **Year** | **Nonrelapse mortality in 1 year (NMR)** | **Transplant related mortality (TRM)** | **One year progression free survival (PFS)** | **Overall Survival 1 year (OS1)** |
| **Svahn** | 2005 |  | Out: 13% In: 44% (Sig) |  | Out: 63% In: 44%, (Sig). At four year´s time |
| **Nicolau** | 2007 | Similar in both groups |  |  |  |
| **Svahn** | 2008 |  |  |  | Out 65% In: 47% (Sig). At five years |
| **McDiarmid** | 2010 | Out: 14.1%, lower (Sig). 100 days |  |  |  |
| **Ringden** | 2013 | Similar in both groups |  |  | Out: 61% In: 49%, (NS). At five years´time |
| **Granot** | 2015 | Out:13% In: 26%, (Sig).  At five years time | |  |  |
| **Guru** | 2019 | Out: 3.2% In: 10.8%, (NS) |  | Out: 63.6% In: 64.4%, (NS) | Out: 82.8% In: 73.8%, (NS). 1 year is assumed |
| **Shingal** | 2020 |  | 5% in-hospital mortality |  |  |
| **Gutierrez-García** | 2020 | Similar in both groups |  |  | Similar in both groups |

NRM: Non Relapse Mortality,

TRM: Transplant Related, Mortality, OS: Overall Survival, PFS: Progression Free Survival, NS: Non Significative and Sig: Significant.

1. González MJ, Urizar E, Urtaran-Laresgoiti M, Nuño-Solinís R, Lázaro-Pérez E, Vázquez L, Pascual-Cascón MJ, Solano C, Kwon M, Gallego C, et al. Hospital and outpatient models for Hematopoietic Stem Cell Transplantation: A systematic review of comparative studies for health outcomes, experience of care and costs. *PLoS One* (2021) 16:1–15. doi: 10.1371/journal.pone.0254135

2. Singhal S, Saadeh SS, Durani U, Kansagra A, Alkhateeb HB, Shah M V., Mangaonkar A, Kenderian S, Hashmi S, Patnaik M V., et al. Allogeneic Hematopoietic Stem Cell Transplantation in the Outpatient Setting: The Mayo Clinic Experience. *Transplant Cell Ther* (2023) 29:183.e1-183.e6. doi: 10.1016/J.JTCT.2022.12.016
