# Supplementary material for: Brave new world: expanding home care in stem cell transplantation and advanced therapies with new technologies
Source: Front Immunol. 2024 Apr 26;15:1366962. doi: 10.3389/fimmu.2024.1366962 (PMC11082320; doi:10.3389/fimmu.2024.1366962)
Supplement: Supplementary file 3 [file Table_3.docx]

| Appendix 3: experiences of CAR-T outpatient management reported at scientific meetings. | | | | |
| --- | --- | --- | --- | --- |
| Author (reference) | Dwivedy (1) | Bachier (2) | Shao (3) | Paludo (4) |
| Year | 2019 | 2020 | 2021 | 2023 |
| Study duration | 1 year | Not stated (*) | 2 years | Not stated |
| Visit schedule after CAR-T infusion | Week 1: days 2 and 4.  Weeks 2 to 4: once a week, starting day 8 | Not stated | Daily, days 1 - 14 | Not stated. Used RPM (**) |
| Patients managed in outpatient setting, n | 28 | 37 | 12 | 123 |
| Type of CAR-T, n | Tisa, 28 | Liso, 37 | Tisa, 12 | Not stated |
| Readmission post CAR-T infusion n (%) | 9 (32%) | 22 (59 %) | 6 (50%) | 104 (84%) |
| Days to readmission after CAR-T infusion, median (range) | 5 (1-7) | 5 (2-22) | 4 (2 -12) | Not stated |
| Days of stay after readmission, median (range) | Not stated | 6 (2-23) | 5.5 (2-9) | 8 (4-27) |
| Adverse events, any grade  CRS n (%)  Neurotoxicity n (%) | 5 (18%)  1 (3.6%) | 16 (43%)  12 (32%) | 6 (50%)  1 (8%) | 92 (75%)  45 (37%) |
| Patients receiving specific treatments for CAR-T adverse effects  Tocilizumab n (%)  Corticosteroids n (%) | Not stated  Not stated | 3 (8%) (§)  3 (8%) (§) | 5 (42%)  1 (8%) | 92 (100%)  63 (68%) |
| ICU or emergency use n (%) | Not stated | 1 (3%) | 2 (33%) | 7 (6%) |
| CAR-T related deaths | None | None | None | Not stated |
| ORR % (type of response) | Not stated | 79 % (14CR, 12PR) | 58.3% (3CR, 4PR) | Not stated |

(*) This study summarizes data from patients who took part in three different clinical trials across several university and non-university hospitals (TRANSCEND NHL 001 (NCT02631044), OUTREACH (NCT03744676), and PILOT (NCT03483103).

(**) all took part in a Remote Patient Monitoring (RPM) scheme to monitor vital signs and neurotoxicity.

(§) Three patients were treated for adverse effects and received both tocilizumab and corticosteroids.

CAR-T: chimeric antigen receptor – T.

Tisa: tisagenlecleucel.

Liso: lisocabtagene maraleucel.

CRS: cytokine release syndrome

ICANS: immune effector cell-associated neurotoxicity syndrome.

ICU: intensive care unit.

ORR: overall response rate. CR: complete remission. PR: partial remission

**REFERENCES**

1. Dwivedy Nasta S, Namoglu EC, Hughes ME, Chong EA, Svoboda J, Ballard HJ, Landsburg DJ, LaRose MI, Barta SK, Gerson JN, et al. Hospitalization Patterns with Commercial CAR T-Cell Therapy: A Single Institution Experience. *Blood* (2019) 134:3240–3240. doi: 10.1182/blood-2019-130650

2. Bachier CR, Palomba ML, Abramson JS, Andreadis C, Sehgal A, Godwin J, Hildebrandt GC, Siddiqi T, Stevens D, Farazi T, et al. Outpatient Treatment with Lisocabtagene Maraleucel (liso-cel) in 3 Ongoing Clinical Studies in Relapsed/Refractory (R/R) Large B Cell Non-Hodgkin Lymphoma (NHL), Including Second-Line Transplant Noneligible (TNE) Patients: Transcend NHL 001, Outreach, and PILOT. *Biology of Blood and Marrow Transplantation* (2020) 26:S25–S26. doi: 10.1016/j.bbmt.2019.12.093

3. Shao YF, Modi D, Kin A, Alavi A, Ayash L, Ratanatharathorn V, Uberti JP, Deol A. Feasibility of Outpatient CAR T Cell Therapy: Experience of a Single Institution. *Blood* (2021) 138:4828–4828. doi: 10.1182/blood-2021-146283

4. Paludo J, Bansal R, Hathcock M, Pritchett JC, De Menezes Silva Corraes A, Oyarzabal BA, Harmsen WS, Lunde JJ, Coffey JD, Haugen KL, et al. Healthcare Utilization Outcomes of Outpatient CAR-T Cell Therapy with Remote Patient Monitoring Program. *Blood* (2023) 142:5075–5075. doi: 10.1182/blood-2023-187834
