# Supplementary material for: Brave new world: expanding home care in stem cell transplantation and advanced therapies with new technologies
Source: Front Immunol. 2024 Apr 26;15:1366962. doi: 10.3389/fimmu.2024.1366962 (PMC11082320; doi:10.3389/fimmu.2024.1366962)
Supplement: Supplementary file 4 [file Table_4.docx]

**Appendix 4. Summary and update of prior published provisional patient inclusion criteria for at-home / OP HSCT or CAR-T therapy programs** (1–4)

| Patient | - Age ≤ 65 years (*) - ECOG ≤ 2 - Written informed consent. - Normal cardiac, lung, liver, and renal function. - Absence of refractoriness to platelet transfusion. - Central venous catheter. - Accepts home transfusion (§). - Secondary prophylaxis for recent documented infection. - Understanding of the process. |
| --- | --- |
| Transplant/therapy Center | - Outpatient clinic available 24 h /day or bed reserved in the transplant unit. - Dedicated phone line 24 h / 365 days to allow patients or their caregivers to contact an expert physician of the transplant team. - Staff trained in outpatient at-home / OP care. |
| Caregiver | - A suitable caregiver available 24 h per day, 7 days a week. - Preferably the same person. - Must undergo training to perform basic chores (detection and communication of warning signs, taking clinical data with automatic devices, safe waste disposal) |
| Home | - Phone line (land or mobile). - Adequate living conditions (electricity, running water, sanitation, well-maintained premises, especially no mold) - Travel time from home to the hospital less than 60 min at rush hour. |

HSCT: hematopoietic stem cell transplantation.

OP: outpatient, where patients receive lymphodepletion / transplant conditioning and infusion in the day clinic or as inpatients and are followed up at an ambulatory day clinic.

CAR-T: chimeric antigen receptor -T

(*) amenable to be changed according to frailty scales.

(§) according to local practice. Could be waived for auto-HSCT.

1. González-Barrera S, Martín-Sánchez G, Parra-Jordán JJ, Fernández-Luis S, Calvo JA, Lobeira R, Yañez L, Manzano A, Carrera C, Baro J, et al. Feasibility of a Hospital-at-Home Program for Autologous Hematopoietic Stem Cell Transplantation. *Transplant Cell Ther* (2023) 29:111.e1-111.e7. doi: 10.1016/J.JTCT.2022.11.018

2. Oluwole OO, Dholaria B, Knight TE, Jain T, Locke FL, Ramsdell L, Nikiforow S, Hashmi H, Mooney K, Bhaskar ST, et al. Chimeric Antigen Receptor T-Cell Therapy in the Outpatient Setting: An Expert Panel Opinion from the American Society for Transplantation and Cellular Therapy. *Transplant Cell Ther* (2023) doi: 10.1016/j.jtct.2023.11.008

3. González MJ, Urizar E, Urtaran-Laresgoiti M, Nuño-Solinís R, Lázaro-Pérez E, Vázquez L, Pascual-Cascón MJ, Solano C, Kwon M, Gallego C, et al. Hospital and outpatient models for Hematopoietic Stem Cell Transplantation: A systematic review of comparative studies for health outcomes, experience of care and costs. *PLoS One* (2021) 16:1–15. doi: 10.1371/journal.pone.0254135

4. Borogovac A, Keruakous A, Bycko M, Holter Chakrabarty J, Ibrahimi S, Khawandanah M, Selby GB, Yuen C, Schmidt S, Autry MT, et al. Safety and feasibility of outpatient chimeric antigen receptor (CAR) T-cell therapy: experience from a tertiary care center. *Bone Marrow Transplant* (2022) 57:1025–1027. doi: 10.1038/s41409-022-01664-z
